# Generation of Vascular Networks from Adipocytes *In Vitro*

**Published:** 2019-08-02

**Authors:** Ana M Blázquez Medela, Ashley Penton, Kristina I Bostrom, Arman Saparov, Medet Jumabay

**Affiliations:** 1 Division of Cardiology, David Geffen School of Medicine at UCLA, USA; 2 Department of Medicine, Nazarbayev University School of Medicine, Kazakhstan

**Keywords:** Adipocytes, Dedifferentiated fat cells, Adult stem cells, Endothelial cells

## Abstract

Multipotent cells derived from white mature adipocytes, referred to as dedifferentiated fat (DFAT) cells have the capacity differentiate into endothelial cells. The objective of this study was to modify the isolation method for DFAT cells in order to optimize the endothelial lineage potential. The adipocytes were preincubated for 24 hours, washed, and then incubated for 5 days to allow the generated DFAT cells to remain in proximity to the adipocytes while the cells aggregated into cell clusters. The DFAT cells rapidly differentiated into adipocytes after which endothelial-like cells (ECs) emerged and formed tube-like structure closely associated with the newly differentiated adipocytes. The lipid-filled cells then gradually disappeared whereas the network of tube structure expanded over the course of 3 weeks. ECs accounted for 35-45% of the cells derived from the DFAT cells, as assessed by qPCR, immunofluorescence and fluorescence-activated cell sorting. The DFAT cell-derived ECs could also be further enriched by magnetic sorting, thereby serving as a mouse cell line for further research.

## Introduction

White mature adipocytes are the most abundant cells within adipose tissue, and the adipose tissue is easy to access as a source of stem cells to generate various types of cells. Specifically, two types of multipotent cells are generated through digestion of adipose tissue; adipose-derived stem cells (ASCs) that are derived from the stromal vascular fraction, and exhibit mesenchymal stem cell properties [1-6] and dedifferentiated fat (DFAT) cells that are derived from the adipocytes, and exhibit similar characteristics but with more homogeneity and may held pluripotent differentiation potential. The DFAT cells have been shown to undergo osteochondrogenic differentiation [7] participate in bone regeneration and re-differentiate into adipocytes [8,9]. In addition, DFAT cells from mice have the ability of spontaneously undergo cardiomyocyte differentiation [10] and endothelial cells (EC] differentiation [11]. The original method for the isolation of DFAT cells consisted of enzymatic digestion of adipose tissue with collagenase followed by isolation of adipocytes and ceiling culture [12]. In this study, we optimize a newly developed isolation method for DFAT cells to enhance endothelial differentiation and show that vascular network can be generated from the adipocytes.

## Methods

### Collection of adipose tissue

Mice on C57BL/6J background were obtained from the Jackson Laboratory. All mice were fed a standard chow diet (Diet 8604; HarlanTeklad Laboratory, Placentia, CA). The studies were reviewed and approved by the Institutional Review Board and conducted in accordance with the animal care guidelines set by the University of California, Los Angeles. The investigation conformed to the standards of the National Research Council [13]. Committee for the Update of the Guide for the Care and Use of Laboratory Animals [13]. The mice were sacrificed with an overdose of isoflurane, and the subcutaneous fat depots were collected under sterile conditions.

### Isolation of adipocytes and culture of DFAT cells

**Figure 1:**
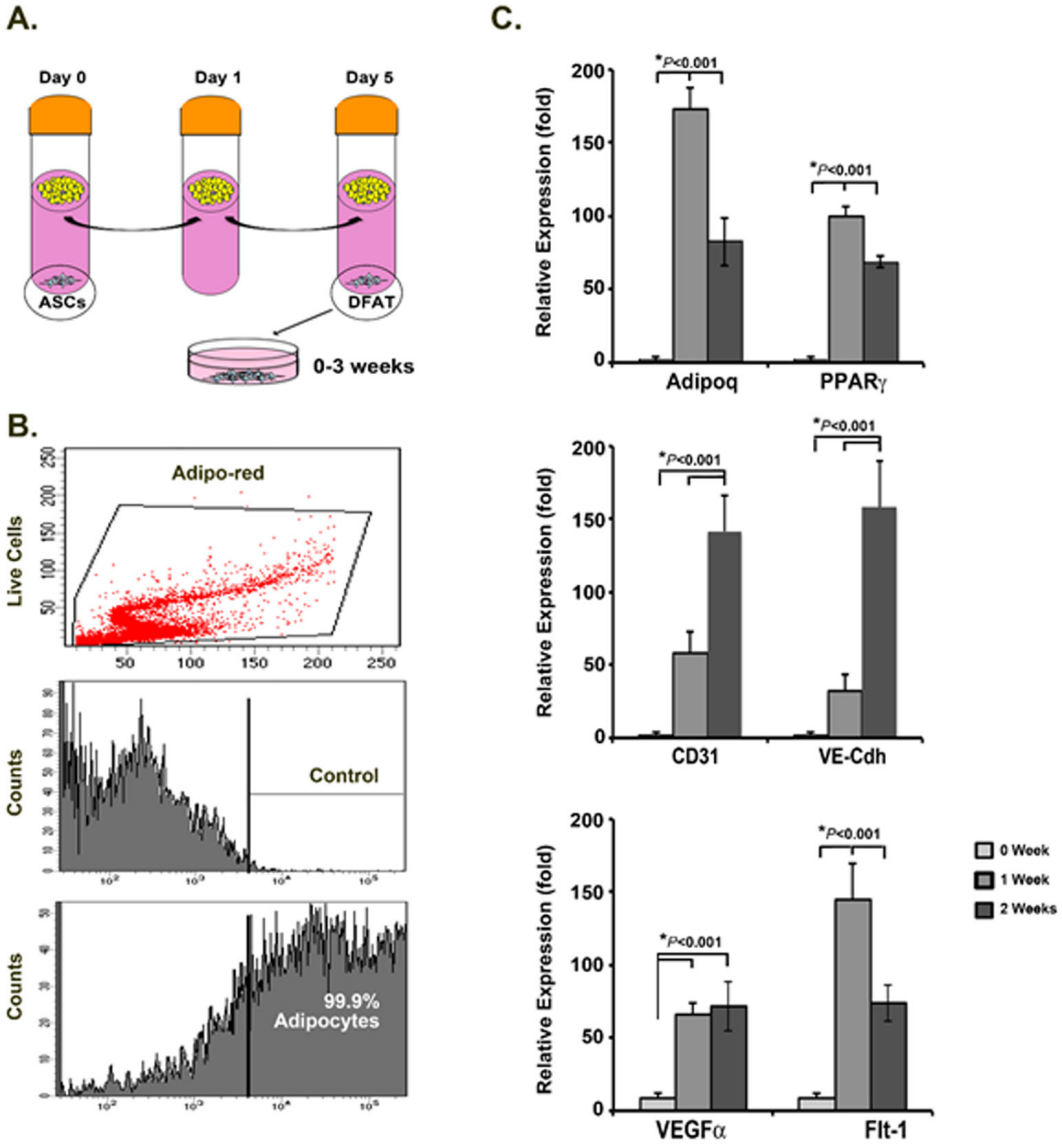
Isolation and differentiation of DFAT cells a modified method. A. The DFAT cells were generated from suspended adipocytes by allowing them sink to the bottom of the tube as illustrated. ASC, adipocyte stromal cell. DFAT, dedifferentiated fat cells. B. Lipid-filled adipocytes were stained with adipo-red and cell purity was assessed by FACS. C. The DFAT cells expressed both adipogenic and angiogenic cell markers as determined by qPCR.

Subcutaneous adipose tissue was minced and digested with collagenase, as previously described [12,14]. The isolated adipocytes were washed 3 times with culture medium, and pre-incubated for 24 hours at 37°C and 5% C02. After the pre-incubation, the adipocytes were washed once again, and then placed in a 50ml tube, with loosened cap, containing 25ml 20%FBS/DMEM (see Figure 1A for schematic illustration of the culture method). On day 5, the cells were centrifuged for 3min at 1000rpm. The floating layer of adipocytes was removed, and the DFAT cells that had formed pellets on the bottom of the tube were collected and distributed at 1x105 cells per well in 12-well dishes and cultured in 20%FBS/DMEM for up to 3 weeks. The medium was changed every 3 days.

## RNA analysis

RNA was collected using the RNeasy Qiagen kit (Qiagen, Carlsbad, CA). Quantitative (q)PCR was performed as previously described [15]. The primers and probes used for qPCR for mouse adiponectin (Adipoq), mouse peroxisome proliferator-activated receptor gamma (PPARγ), rat CCAAT/enhancer-binding protein alpha (C/EBPα)), mouse cluster of differentiation (CD) 31, mouse vascular endothelial growth factor (VEGF), mouse VEGF receptor 2 (VEGFR2), mouse CD34 and mouse VE-Cadherin (VE-Cdh) were pre-designed and obtained from Applied Biosystems (Foster City, CA) as part of Taqman® Gene Expression Assays.

### Immunohistochemistry and immunocytochemistry

Cells grown in chamber slides were fixed in 4% paraformaldehyde, permeabilized with 0.2% Triton X-100, blocked with 10% goat serum and 1% BSA in phosphate-buffered saline (PBS), and incubated over night at 4ºC with the appropriate primary antibodies (anti-perilipin, anti-CD31) or non-specific IgG control antibodies, diluted 1:200 in 1% BSA in PBS. The next day, cells were incubated with secondary AF-488-conjugated (green fluorescence) or AF-594-conjugated (red fluorescence) goat anti-mouse or anti-rabbit secondary antibodies (Thermo Fisher, Waltham, MA). The cells were washed with PBS, the nuclei stained with 4’,6-diamidino-2-phenylindole (DAPI, Sigma-Aldrich, Saint Louis, MO), and visualized by fluorescence microscopy.

## Flow Cytometric Analysis

The purity and size of the isolated adipocytes was assessed by fluorescence-activated cell sorting (FACS) using the lipophilic fluorescent dye Adipo Red as previously described [16,17]. For characterization of the phenotype of endothelial-like cells, FACS analysis was performed after 21 days of culture as previously described [16]. using fluorescein isothiocyanate (FITC)-, or phycoerythrin (PE)- conjugated anti-mouse antibodies against CD31 and CD34.

### Time lapse and linage tracing

The cells were cultured as described above and samples for RNA analysis were collected on days 0, 3, 5, 7, 14 and 21. On the same days, live microscopy pictures of the cells were taken, and immunofluorescence was performed.

### Isolation and staining of retina

Retinas were isolated as previously described [18]. In brief: eyes were collected from wild-type C57BL/6J mice at 7 days of age, and briefly fixed for 20 min in 4% paraformaldehyde (PFA) with 2x phosphate buffered saline (PBS). Retinas were dissected from the sclera and choroid in PBS and clipped to unfold into a clover-like structure, followed by an additional fixation with cold methanol for 24 hours. After this fixation, retinas were washed 3 times with PBS for 10 minutes. Afterwards, retinas were incubated in blocking solution (0.1%Triton X-100, 1% fetal bovine serum (FBS) in PBS for 1-3 hours at room temperature. The samples were incubated overnight at 4°C with Griffonia simplicifolia isolectin B4 (1:200 dilution, Vector Labs, Burlingame, CA), a preconjugated glycoprotein that recognizes sugars on the surface of endothelial cells and enables visualization of blood vessels. The samples were subsequently washed in PBS containing 0.1% Triton four times for 30 min each and mounted using Vectashield (Vector Labs, Burlingame, CA).

## Statistical Analysis

Data were analyzed for statistical significance by two-way analysis of variance with post hoc Tukey’s analysis using the GraphPad Prism 3.0 software (GraphPad Software, La Jolla, CA). P values less than 0.05 were considered significant. All experiments were repeated a minimum tree time.

## Results

### Adipocyte isolation and dedifferentiation

To optimize the EC lineage potential in the DFAT cells, we prolonged the exposure time of the DFAT cells to the adipocytes in part mimicking exposure to hypoxic adipocytes in tissue. The DFAT cells were prepared as described in the method section. The ASCs were not included in the present study. The purity of the initial adipocytes was 99.9% as determined by AdipoRed-staining and FACS analysis (Figure 1B). The results from qPCR indicated that the gene signature of both adipocytes and endothelial cells first emerged in the cell pellet on the bottom of the tube. Expression of the adipocyte markers PPARγ and Adipoq increased in the first week but decreased in the second week after plating whereas the endothelial markers CD31 and VE-cadherin increased in the second and third week of cell differentiation (Figure 1C). The angiogenic marker VEGF and its receptor Flt1 also increased progressively after the first week of culture, suggesting that adipogenic re-differentiation preceded endothelial differentiation.

### Adipocyte and endothelial-like differentiation of DFAT cells

**Figure 2:**
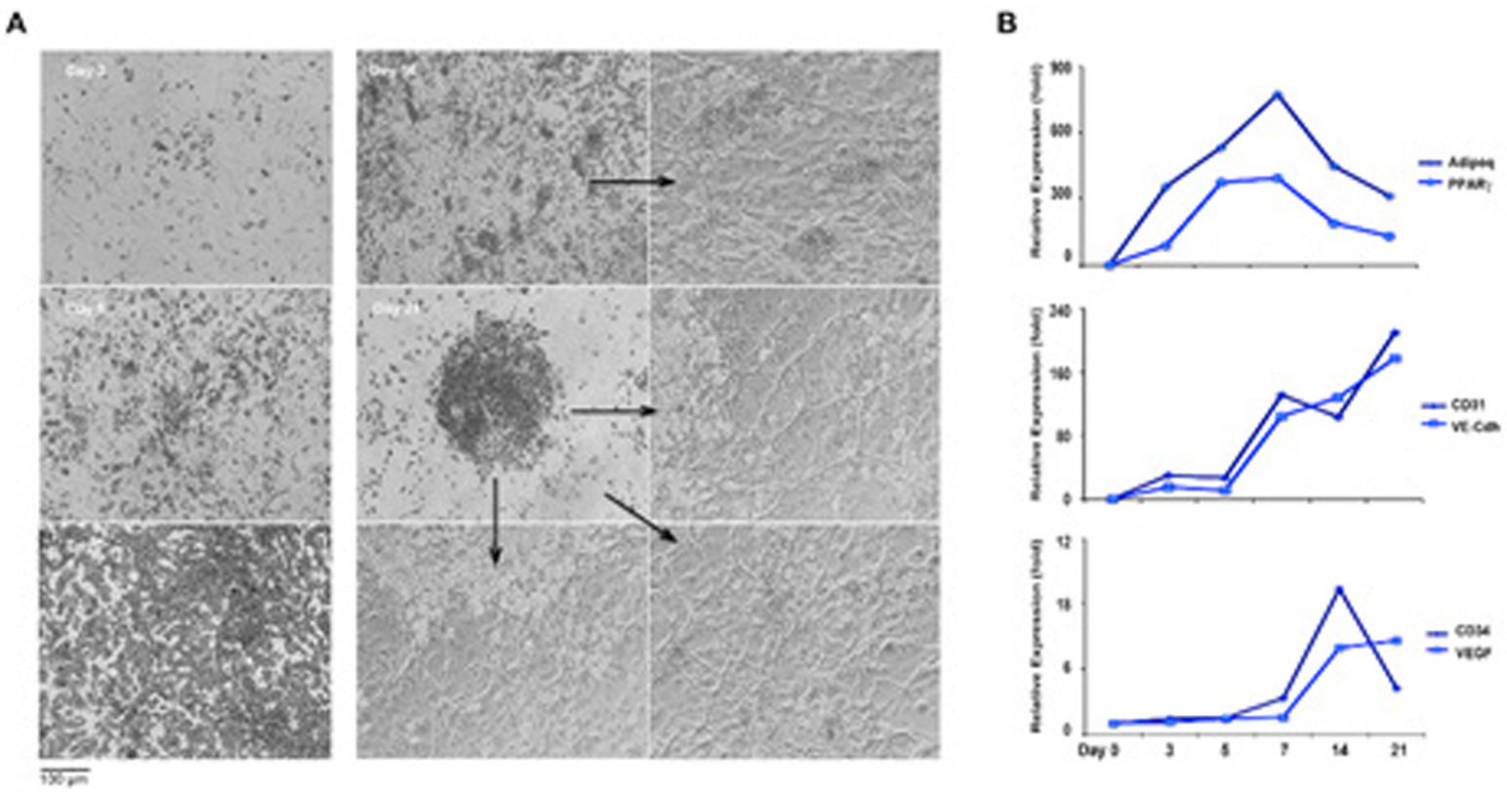
Time course of DFAT cell differentiation after use of the modified method. A. Bright field images demonstrated morphologic changes in the DFAT cells between day 3 to day 21. B. Changes in expression during the time course of adipocyte and endothelial markers.

In order to examine the relationship between adipocytes and EC-like cells derived from DFAT cells, we performed time course experiments without passaging the cells. We observed that cells with lipid droplets emerged as early as after 3-5 days and were most abundant on day 7. Interestingly, the lipid-filled cells gradually decreased after 10 days of culture. Meanwhile, during the same period, networks of EC-like cells emerged, sprouting from under clusters of lipid filled cells (Figure 2A). The qPCR result showed that expression levels of endothelial and adipocyte markers coincided with the cellular changes. The adipocyte markers Adipoq and PPARγ increased dramatically during the first 3 to 7 days and decreased after 10 to 21 days (Figure 2B). On the other hand, the endothelial markers CD31 and VE-cadherin emerged on day 7 and kept increasing on day 10 to 21. The additional endothelial markers VEGF and Flt1 increased at the same time as the tubular structures emerged (Figure 2B). The results suggested that the DFAT cells obtained with the modified method had a high differentiation potential for both adipogenic and endothelial lineages. Moreover, the finding that adipocyte-like cells emerged first, followed by EC-like cells differentiated later, suggested a close relationship between adipocytes and endothelial cells.

### Tubular formation of endothelial-like cells

**Figure 3:**
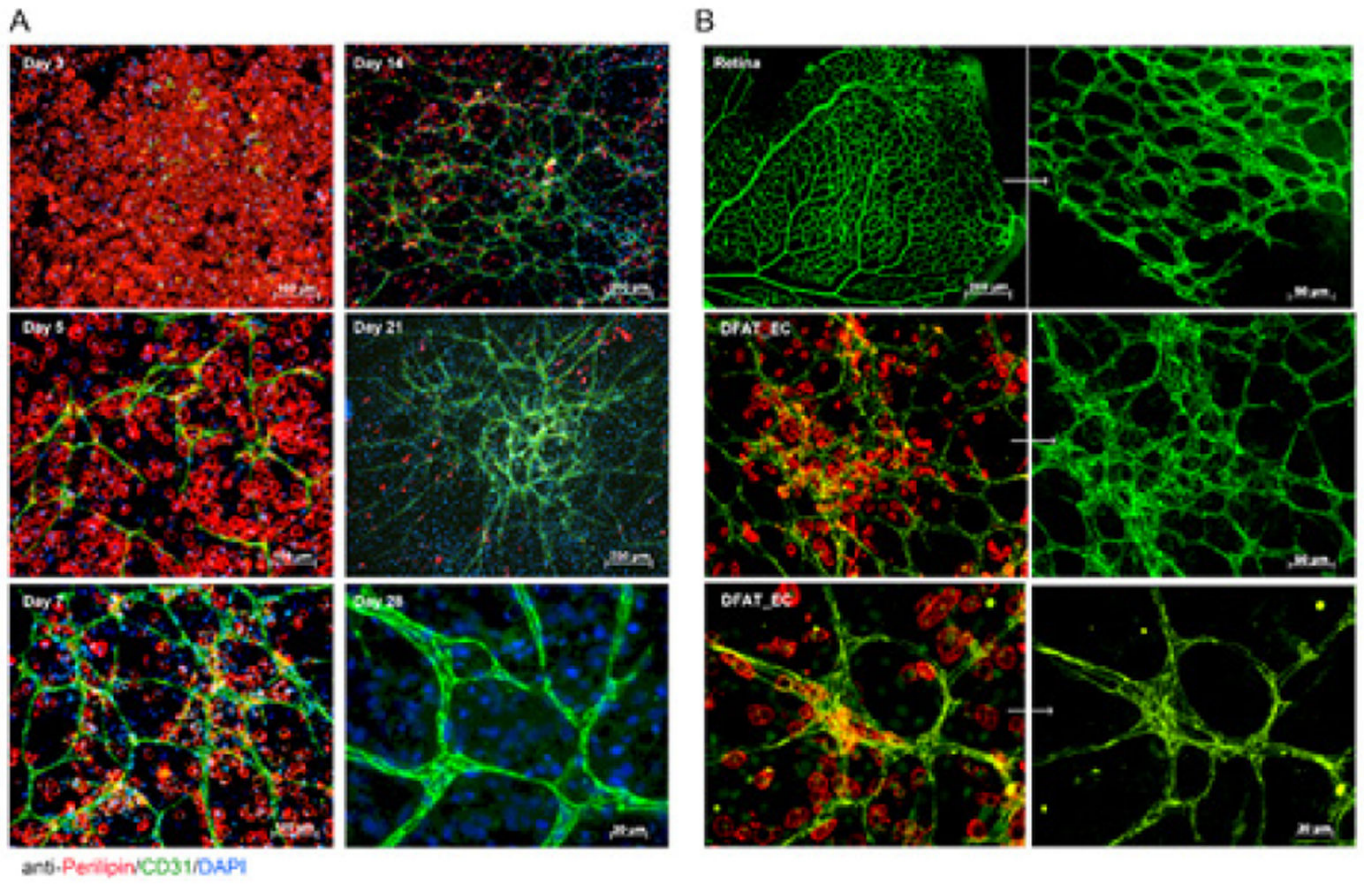
Immuno-staining against Perilipin (red), and CD31 (green). Perilipin was highly expressed in the cells on day 3 and day 5. A. Tubular structures expressed CD31 at day 7 and was enriched network on day 21. B. Capillary networks in retina stained for CD31 and were comparable to tubular network in DFAT cells.

In order to assess the maturity of the EC-like cells derived from the DFAT cells, we examined tubular structures formed by the cells using immunostaining and compared them with vascular network in postnatal mouse retinas. CD31 positive cells increased on day 5 and started to form tubular structures (Figure 3A). On day 7 to 14 the networks matured and became highly connected and the density of tubular structures increased. To compare the *in vitro* networks with networks in vivo, we isolated and immunostained postnatal mouse retinas. The immunostaining for CD31 showed that the network formation of DFAT cells was similar to the capillary networks in the postnatal mouse retina (Figure 3B), supporting the endothelial nature of the differentiated DFAT cells. In addition, we obtained time-lapse monitoring of the cell activity during differentiation (supplementary video). The results showed that the DFAT cells formed adipocyte-like clusters during the first 3-5 days. Subsequently, tube-like structures formed vascular networks, supporting endothelial functionality and maturity of the cells.

### Quantification endothelial-like cells

**Figure 4:**
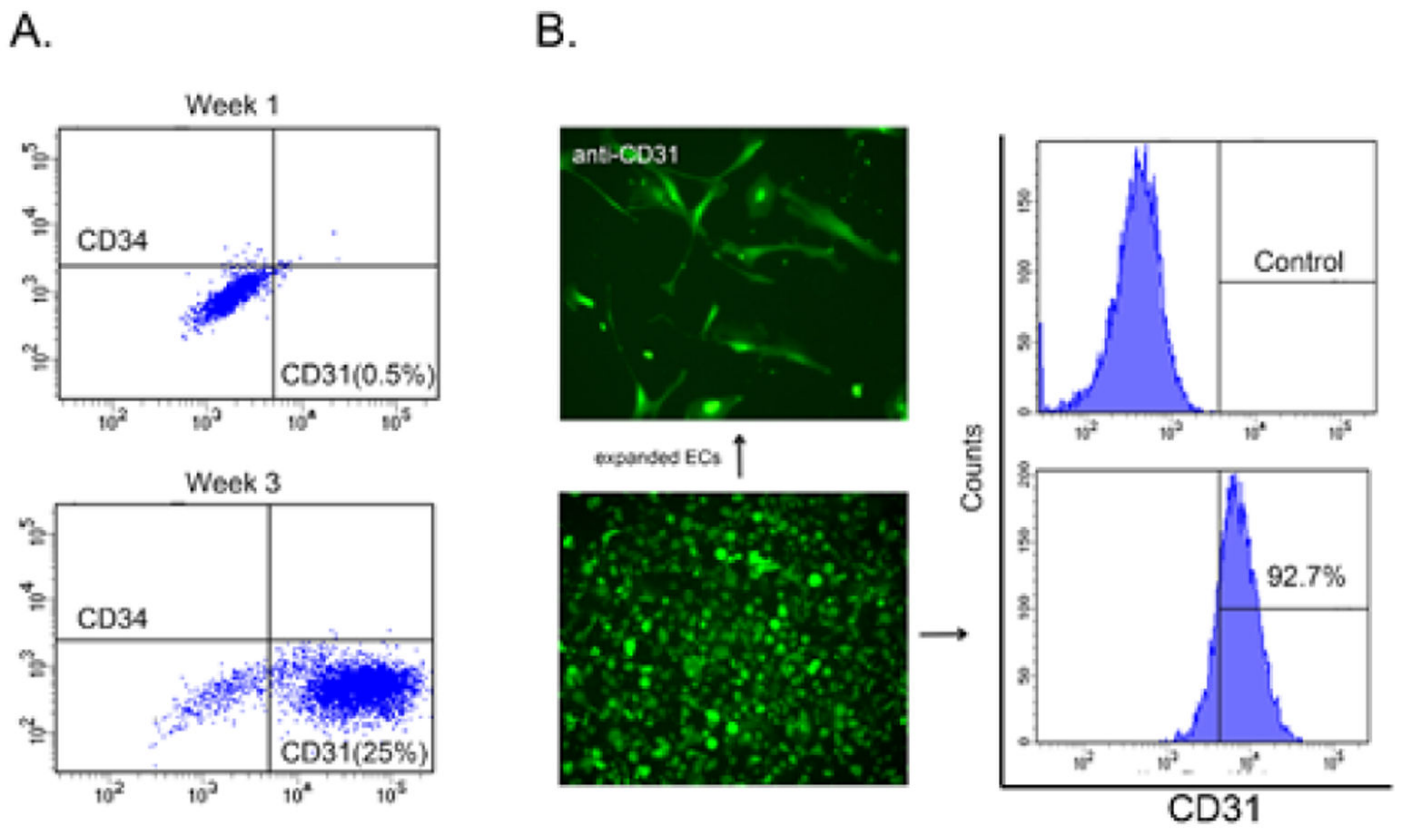
Fluorescence-activated cell sorting (FACS) results. A. Number of CD31 positive cells increased in DFAT cells obtained through tubular method. B. The CD31 positive cells were selected using magnet beads, and expression was shown in counts.

To quantify the ECs derived from DFAT cells using the modified method, we performed FACS analysis after 21 days of culture. The result showed that the percent of CD31 positive cells in the total cell population increased from 0.5 to 25% (Figure 4A). CD34 positive cells, which represented endothelial progenitor cell decreased (Figure 4A). We were able to recover a total of 92.7% of CD31 positive cells from fluorescent magnet beads and culture them in EC medium (Figure 4B). The cells survived and proliferated suggesting that they may be used as an EC cell line for studies of endothelial regeneration. Altogether, our results show that modification in the DFAT cell preparation may alter the characteristics of the cells and increase its lineage potential toward specific cell types such as endothelial cells.

## Discussion

Our data show that the EC-like cells that were derived from the DFAT cells using our modified method resembled ECs in their shape, expressed endothelial markers and were able to form vascular networks that resembled those typically found in mouse retinas. These vascular networks developed spontaneously, without the addition of any inducing agent, which suggests that this culture method could be used to study the mechanisms that trigger and control the development of vascular networks. The main difference between this modified method of DFAT culture with respect to the previous ones is that we allow the presence of adipocytes in culture for a longer time. Thus, the adipocytes present in the culture may be responsible for enhancing endothelial differentiation.

Some studies have previously described the ability of adipose tissue to secrete adipokines and angiogenesis inhibitors that can modulate vessel formation, such as adiponectin [19,20], plasminogen activator inhibitor [21,22] and resistin [23]. Adipose tissue can also release pro-angiogenic factors, such as VEGF-A [24,25], VEGF-B [26], VEGF-C [27] and angiopoietin 1 and 2 [28,29]. In addition, some of the lipids secreted by adipocytes, such as monobutyrin may have pro-angiogenic effects [30]. Moreover, the accumulation of adipocytes may lead to mild hypoxia and it is known that cells under hypoxic conditions release hypoxia-inducible factor (HIF-1α), which has the ability to induce BMP4 and chordin-like 1 [31,32] that are able to regulate endothelial differentiation, proliferation and angiogenesis [31,33-35]. Further studies are needed to verify this hypothesis, and to examine in detail the possibilities of gradients of HIF-1α and BMP4, as well as other possible factors released by the adipocytes, being in control of the development of the vascular network.

Our results point to the possibility that adipocytes trigger and drive angiogenesis, at least *in vitro* and possibly in the adipose tissue. Several authors have proposed targeting angiogenesis regulation as a way to combat obesity and there are some studies that support this possibility [36-38], although there is some controversy on whether the effects on obesity are direct effects of modification in angiogenesis or not. Nevertheless, our new method can be used to further explore this topic and could potentially yield results with translational applications. Our new culture method also yields a higher percentage of EC cells than previous methods [11,12]. Combining this method with the use of magnetic beads to isolate ECs would be an efficient way to obtain of ECs. Additionally, our new culture method is a promising model for the study of vascular networks development *in vitro* that would complement the already existent methods by focusing on the ability of adipocytes to influence angiogenesis.

## Supplementary Material

SupplementalVideo
